# Crystal structure of 2-[bis(1*H*-pyrazol-1-yl)meth­yl]pyridine

**DOI:** 10.1107/S2056989015013195

**Published:** 2015-07-15

**Authors:** Kyung-sun Son, Jong-Eun Park, Daeyoung Kim, Sung Kwon Kang

**Affiliations:** a Department of Chemistry, Chungnam National University, Daejeon 305-764, Republic of Korea

**Keywords:** crystal structure, pyrazol­yl, pyrid­yl, C—H⋯N inter­actions, crystal structure

## Abstract

The title compound, C_12_H_11_N_5_, was synthesized as a potential tridentate ligand to make catalytic metal complexes. The dihedral angle between the pyrazolyl rings is 67.9 (1)°. The most prominent feature in the crystal packing are C—H⋯N hydrogen-bonding inter­actions that link the mol­ecules into a supra­molecular tape along the *b-*axis direction.

## Related literature   

For the synthesis of the title compound, see: Park *et al.* (2015 ▸); Hoffmann *et al.* (2010 ▸). For metal complexes of the similar ligands, see: Anderson *et al.* (2000 ▸); Liu *et al.* (2011 ▸); Xiao *et al.* (2012 ▸). For potential applications of similar ligands in catalysis, see: Park *et al.* (2015 ▸); Zhang *et al.* (2009 ▸).
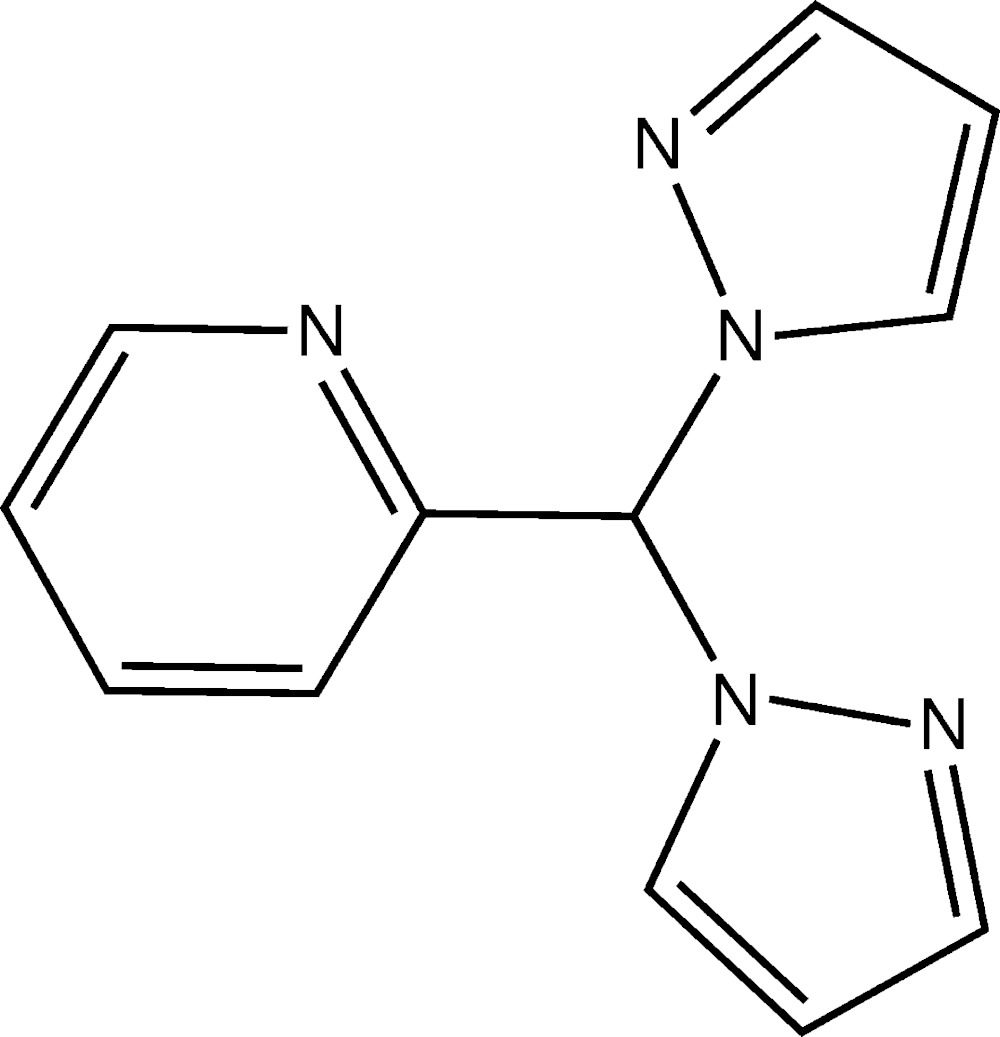


## Experimental   

### Crystal data  

C_12_H_11_N_5_
*M* *_r_* = 225.26
Triclinic, 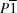

*a* = 7.5723 (3) Å
*b* = 8.6376 (3) Å
*c* = 9.7354 (5) Å
α = 97.539 (2)°
β = 106.123 (4)°
γ = 105.510 (5)°
*V* = 574.73 (5) Å^3^
*Z* = 2
Mo *K*α radiation
μ = 0.09 mm^−1^
*T* = 173 K
0.26 × 0.24 × 0.09 mm

### Data collection  

Bruker SMART CCD area-detector diffractometer
18045 measured reflections
2870 independent reflections
1813 reflections with *I* > 2σ(*I*)
*R*_int_ = 0.089

### Refinement  

*R*[*F* ^2^ > 2σ(*F* ^2^)] = 0.047
*wR*(*F* ^2^) = 0.123
*S* = 0.98
2870 reflections
153 parameters
H-atom parameters constrained
Δρ_max_ = 0.32 e Å^−3^
Δρ_min_ = −0.26 e Å^−3^

### 

Data collection: *SMART* (Bruker, 2002 ▸); cell refinement: *SAINT* (Bruker, 2002 ▸); data reduction: *SAINT*; program(s) used to solve structure: *SHELXS2013* (Sheldrick, 2008 ▸); program(s) used to refine structure: *SHELXL2013* (Sheldrick, 2015 ▸); molecular graphics: *ORTEP-3 for Windows* (Farrugia, 2012 ▸); software used to prepare material for publication: *publCIF* (Westrip,2010 ▸).

## Supplementary Material

Crystal structure: contains datablock(s) I, New_Global_Publ_Block. DOI: 10.1107/S2056989015013195/tk5373sup1.cif

Structure factors: contains datablock(s) I. DOI: 10.1107/S2056989015013195/tk5373Isup2.hkl

Supporting information file. DOI: 10.1107/S2056989015013195/tk5373Isup3.cml

**Fig. 1.**
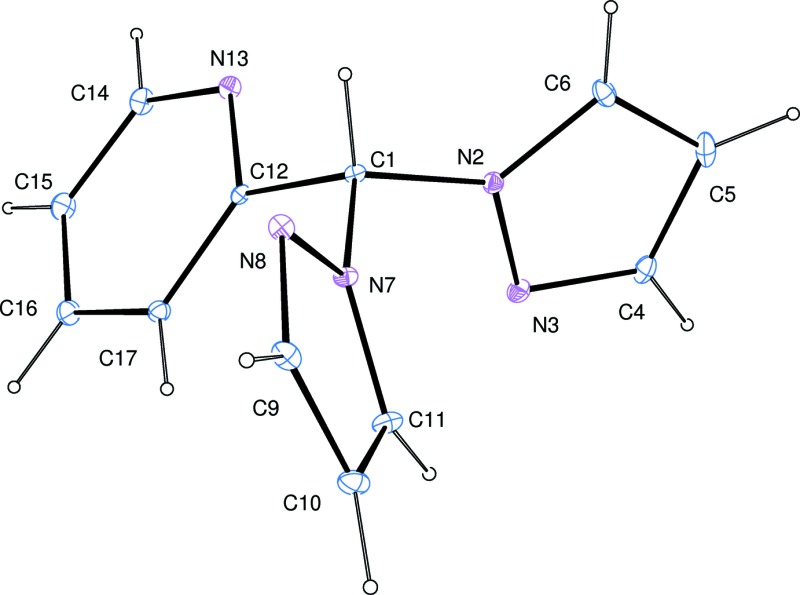
Molecular structure of the title compound, showing the atom-numbering scheme and 30% probability displacement ellipsoids.

**Fig. 2.**
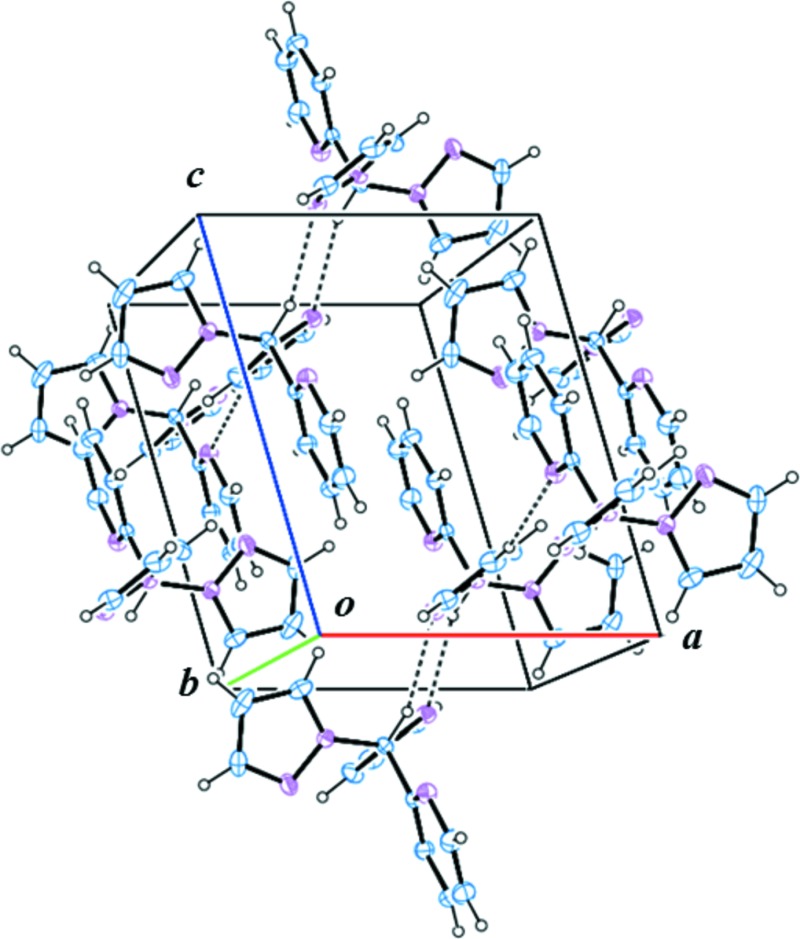
Part of the crystal structure of the title compound, showing supramolecular tapes aligned along the b axis and sustained by C—H···N hydrogen bonds (dashed lines).

. DOI: 10.1107/S2056989015013195/tk5373fig1.tif
Mol­ecular structure of the title compound, showing the atom-numbering scheme and 30% probability displacement ellipsoids.

b . DOI: 10.1107/S2056989015013195/tk5373fig2.tif
Part of the crystal structure of the title compound, showing supra­molecular tapes aligned along the *b* axis and sustained by C—H⋯N hydrogen bonds (dashed lines).

CCDC reference: 1411603

Additional supporting information:  crystallographic information; 3D view; checkCIF report

## Figures and Tables

**Table 1:** Hydrogen-bond geometry (, )

*D*H*A*	*D*H	H*A*	*D* *A*	*D*H*A*
C1H1N8^i^	0.98	2.45	3.3974(18)	162
C10H10N13^ii^	0.93	2.60	3.496(2)	161

Symmetry codes: (i) 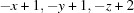; (ii) 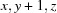.

## supplementary crystallographic information

### S1.1. Synthesis and crystallization

In a 50 ml Schlenk flask, NaH (0.24 g, 10 mmol) was added in dry tetra­hydro­furan (THF; 10 ml) and stirred at 0 °C. Pyrazole (0.68 g, 10 mmol) was added gradually to the mixture over 10 min. and the stirring was continued for 40 min. at 0 °C, resulting in a pale-yellow solution. Thio­nyl chloride (0.38 mL, 5 mmol) was added drop wise to this mixture at 0 °C. After stirring for 1 h, pyridine-2-aldehyde (0.48 ml, 5 mmol) and a catalytic amount of cobalt(II) chloride were added and the resulting solution was refluxed overnight. The reaction mixture was allowed to cool to room temperature, and di­ethyl ether and water (1:1) were added. The bi-phasic solution was stirred for 45 min. to quench the cobalt catalyst. The aqueous layer was extracted three times with di­ethyl ether. The combined organic layers were dried over sodium sulfate and filtered. The solvent in the filtrate was removed *in vacuo* and the resulting solid was purified by column chromatography on silica gel, with ethyl acetate as the eluent. An off-white solid was obtained in 33% yield. Single crystals of the title compound were obtained by slow diffusion of hexane into a concentrated solution of the product in THF at room temperature.

### S1.2. Refinement

All H atoms were positioned geometrically and refined using riding model, with d(C—H) = 0.93–0.98 Å, and with *U*_iso_(H) = 1.2*U*_eq_(C).

### Crystal data

**Table:**

C_12_H_11_N_5_	*Z* = 2
*M**_r_* = 225.26	*F*(000) = 236
Triclinic, *P*1	*D*_x_ = 1.302 Mg m^−^^3^
*a* = 7.5723 (3) Å	Mo *K*α radiation, λ = 0.71073 Å
*b* = 8.6376 (3) Å	Cell parameters from 2704 reflections
*c* = 9.7354 (5) Å	θ = 2.2–22.9°
α = 97.539 (2)°	µ = 0.09 mm^−^^1^
β = 106.123 (4)°	*T* = 173 K
γ = 105.510 (5)°	Plate, colourless
*V* = 574.73 (5) Å^3^	0.26 × 0.24 × 0.09 mm

### Data collection

**Table:**

Bruker SMART CCD area-detector diffractometer	*R*_int_ = 0.089
Radiation source: fine-focus sealed tube	θ_max_ = 28.4°, θ_min_ = 2.2°
φ and ω scans	*h* = −10→10
18045 measured reflections	*k* = −11→11
2870 independent reflections	*l* = −12→13
1813 reflections with *I* > 2σ(*I*)	

### Refinement

**Table:**

Refinement on *F*^2^	0 restraints
Least-squares matrix: full	Hydrogen site location: inferred from neighbouring sites
*R*[*F*^2^ > 2σ(*F*^2^)] = 0.047	H-atom parameters constrained
*wR*(*F*^2^) = 0.123	*w* = 1/[σ^2^(*F*_o_^2^) + (0.0585*P*)^2^] where *P* = (*F*_o_^2^ + 2*F*_c_^2^)/3
*S* = 0.98	(Δ/σ)_max_ < 0.001
2870 reflections	Δρ_max_ = 0.32 e Å^−^^3^
153 parameters	Δρ_min_ = −0.26 e Å^−^^3^

### Special details

**Table:**

Geometry. All e.s.d.'s (except the e.s.d. in the dihedral angle between two l.s. planes) are estimated using the full covariance matrix. The cell e.s.d.'s are taken into account individually in the estimation of e.s.d.'s in distances, angles and torsion angles; correlations between e.s.d.'s in cell parameters are only used when they are defined by crystal symmetry. An approximate (isotropic) treatment of cell e.s.d.'s is used for estimating e.s.d.'s involving l.s. planes.

### Fractional atomic coordinates and isotropic or equivalent isotropic displacement parameters (Å^2^)

**Table:**

	*x*	*y*	*z*	*U*_iso_*/*U*_eq_	
C1	0.2525 (2)	0.40769 (18)	0.78430 (15)	0.0249 (3)	
H1	0.3386	0.3778	0.8644	0.030*	
N2	0.05923 (18)	0.35498 (16)	0.79587 (13)	0.0289 (3)	
N3	−0.09589 (19)	0.34989 (18)	0.68237 (15)	0.0373 (4)	
C4	−0.2452 (3)	0.3038 (2)	0.7301 (2)	0.0421 (4)	
H4	−0.3725	0.2901	0.6753	0.051*	
C5	−0.1886 (3)	0.2781 (2)	0.8731 (2)	0.0488 (4)	
H5	−0.2673	0.2452	0.9292	0.059*	
C6	0.0106 (3)	0.3128 (2)	0.91191 (18)	0.0382 (4)	
H6	0.0940	0.3078	1.0004	0.046*	
N7	0.32364 (18)	0.58610 (15)	0.80514 (12)	0.0268 (3)	
N8	0.50866 (18)	0.66766 (16)	0.89361 (13)	0.0311 (3)	
C9	0.5303 (3)	0.8249 (2)	0.89025 (18)	0.0370 (4)	
H9	0.6438	0.9111	0.9418	0.044*	
C10	0.3645 (3)	0.8456 (2)	0.80116 (18)	0.0420 (5)	
H10	0.3457	0.9437	0.7819	0.050*	
C11	0.2343 (3)	0.6900 (2)	0.74763 (18)	0.0382 (4)	
H11	0.1080	0.6612	0.6837	0.046*	
C12	0.2556 (2)	0.31567 (18)	0.64180 (15)	0.0237 (3)	
N13	0.24020 (19)	0.15718 (16)	0.64034 (14)	0.0323 (3)	
C14	0.2372 (3)	0.0666 (2)	0.51714 (19)	0.0405 (4)	
H14	0.2239	−0.0441	0.5137	0.049*	
C15	0.2520 (3)	0.1246 (2)	0.3968 (2)	0.0456 (5)	
H15	0.2493	0.0558	0.3138	0.055*	
C16	0.2709 (3)	0.2866 (2)	0.40071 (18)	0.0421 (4)	
H16	0.2829	0.3305	0.3202	0.050*	
C17	0.2721 (2)	0.3854 (2)	0.52497 (16)	0.0326 (4)	
H17	0.2837	0.4960	0.5294	0.039*	

### Atomic displacement parameters (Å^2^)

**Table:**

	*U*^11^	*U*^22^	*U*^33^	*U*^12^	*U*^13^	*U*^23^
C1	0.0240 (8)	0.0233 (8)	0.0262 (7)	0.0089 (6)	0.0048 (6)	0.0061 (6)
N2	0.0294 (8)	0.0300 (7)	0.0288 (7)	0.0111 (6)	0.0106 (6)	0.0055 (5)
N3	0.0264 (8)	0.0442 (9)	0.0416 (8)	0.0144 (7)	0.0078 (6)	0.0116 (7)
C4	0.0279 (9)	0.0368 (10)	0.0615 (12)	0.0103 (8)	0.0176 (8)	0.0040 (8)
C5	0.0579 (13)	0.0362 (10)	0.057	0.0060 (9)	0.0400 (8)	−0.0013 (9)
C6	0.0504 (12)	0.0311 (10)	0.0332 (9)	0.0075 (8)	0.0206 (8)	0.0036 (7)
N7	0.0300 (7)	0.0242 (7)	0.0237 (6)	0.0098 (6)	0.0043 (5)	0.0039 (5)
N8	0.0312 (8)	0.0285 (8)	0.0274 (7)	0.0044 (6)	0.0069 (6)	0.0018 (5)
C9	0.0483 (11)	0.0250 (9)	0.0334 (8)	0.0043 (8)	0.0155 (8)	0.0023 (7)
C10	0.0662 (13)	0.0265 (9)	0.0376 (9)	0.0196 (9)	0.0186 (9)	0.0082 (7)
C11	0.0457 (11)	0.0321 (9)	0.0368 (9)	0.0210 (8)	0.0053 (8)	0.0078 (7)
C12	0.0180 (8)	0.0244 (8)	0.0268 (7)	0.0076 (6)	0.0049 (6)	0.0029 (6)
N13	0.0332 (8)	0.0259 (7)	0.0347 (7)	0.0081 (6)	0.0103 (6)	0.0018 (6)
C14	0.0416 (11)	0.0320 (10)	0.0421 (10)	0.0088 (8)	0.0128 (8)	−0.0035 (8)
C15	0.0471 (12)	0.0455 (12)	0.0410 (10)	0.0153 (9)	0.0141 (8)	−0.0009 (8)
C16	0.0478 (11)	0.0506 (12)	0.0324 (9)	0.0205 (9)	0.0162 (8)	0.0076 (8)
C17	0.0371 (10)	0.0318 (9)	0.0321 (8)	0.0152 (8)	0.0119 (7)	0.0076 (7)

### Geometric parameters (Å, º)

**Table:**

C1—N2	1.4527 (19)	C9—C10	1.384 (2)
C1—N7	1.4559 (18)	C9—H9	0.9300
C1—C12	1.515 (2)	C10—C11	1.368 (2)
C1—H1	0.9800	C10—H10	0.9300
N2—C6	1.346 (2)	C11—H11	0.9300
N2—N3	1.3570 (17)	C12—N13	1.3403 (19)
N3—C4	1.323 (2)	C12—C17	1.375 (2)
C4—C5	1.404 (3)	N13—C14	1.334 (2)
C4—H4	0.9300	C14—C15	1.354 (3)
C5—C6	1.386 (2)	C14—H14	0.9300
C5—H5	0.9300	C15—C16	1.362 (3)
C6—H6	0.9300	C15—H15	0.9300
N7—C11	1.3502 (19)	C16—C17	1.382 (2)
N7—N8	1.3578 (16)	C16—H16	0.9300
N8—C9	1.330 (2)	C17—H17	0.9300
			
N2—C1—N7	110.59 (12)	N8—C9—H9	123.9
N2—C1—C12	110.54 (12)	C10—C9—H9	123.9
N7—C1—C12	113.54 (12)	C11—C10—C9	104.88 (15)
N2—C1—H1	107.3	C11—C10—H10	127.6
N7—C1—H1	107.3	C9—C10—H10	127.6
C12—C1—H1	107.3	N7—C11—C10	107.06 (15)
C6—N2—N3	112.85 (14)	N7—C11—H11	126.5
C6—N2—C1	127.42 (13)	C10—C11—H11	126.5
N3—N2—C1	119.70 (12)	N13—C12—C17	122.77 (14)
C4—N3—N2	104.30 (14)	N13—C12—C1	113.00 (12)
N3—C4—C5	112.05 (16)	C17—C12—C1	124.22 (13)
N3—C4—H4	124.0	C14—N13—C12	116.65 (14)
C5—C4—H4	124.0	N13—C14—C15	124.63 (17)
C6—C5—C4	104.55 (16)	N13—C14—H14	117.7
C6—C5—H5	127.7	C15—C14—H14	117.7
C4—C5—H5	127.7	C14—C15—C16	118.08 (17)
N2—C6—C5	106.26 (15)	C14—C15—H15	121.0
N2—C6—H6	126.9	C16—C15—H15	121.0
C5—C6—H6	126.9	C15—C16—C17	119.63 (16)
C11—N7—N8	111.66 (13)	C15—C16—H16	120.2
C11—N7—C1	130.08 (13)	C17—C16—H16	120.2
N8—N7—C1	118.25 (11)	C12—C17—C16	118.22 (15)
C9—N8—N7	104.15 (13)	C12—C17—H17	120.9
N8—C9—C10	112.24 (15)	C16—C17—H17	120.9

### Hydrogen-bond geometry (Å, º)

**Table:**

*D*—H···*A*	*D*—H	H···*A*	*D*···*A*	*D*—H···*A*
C1—H1···N8^i^	0.98	2.45	3.3974 (18)	162
C10—H10···N13^ii^	0.93	2.60	3.496 (2)	161

Symmetry codes: (i) −*x*+1, −*y*+1, −*z*+2; (ii) *x*, *y*+1, *z*.
