# Supplementary figures and images for: Crystal structure of 2-[bis(1H-pyrazol-1-yl)meth­yl]pyridine
Source: Acta Crystallogr E Crystallogr Commun. 2015 Jul 15;71(Pt 8):o567. doi: 10.1107/S2056989015013195 (PMC4571400; doi:10.1107/S2056989015013195)

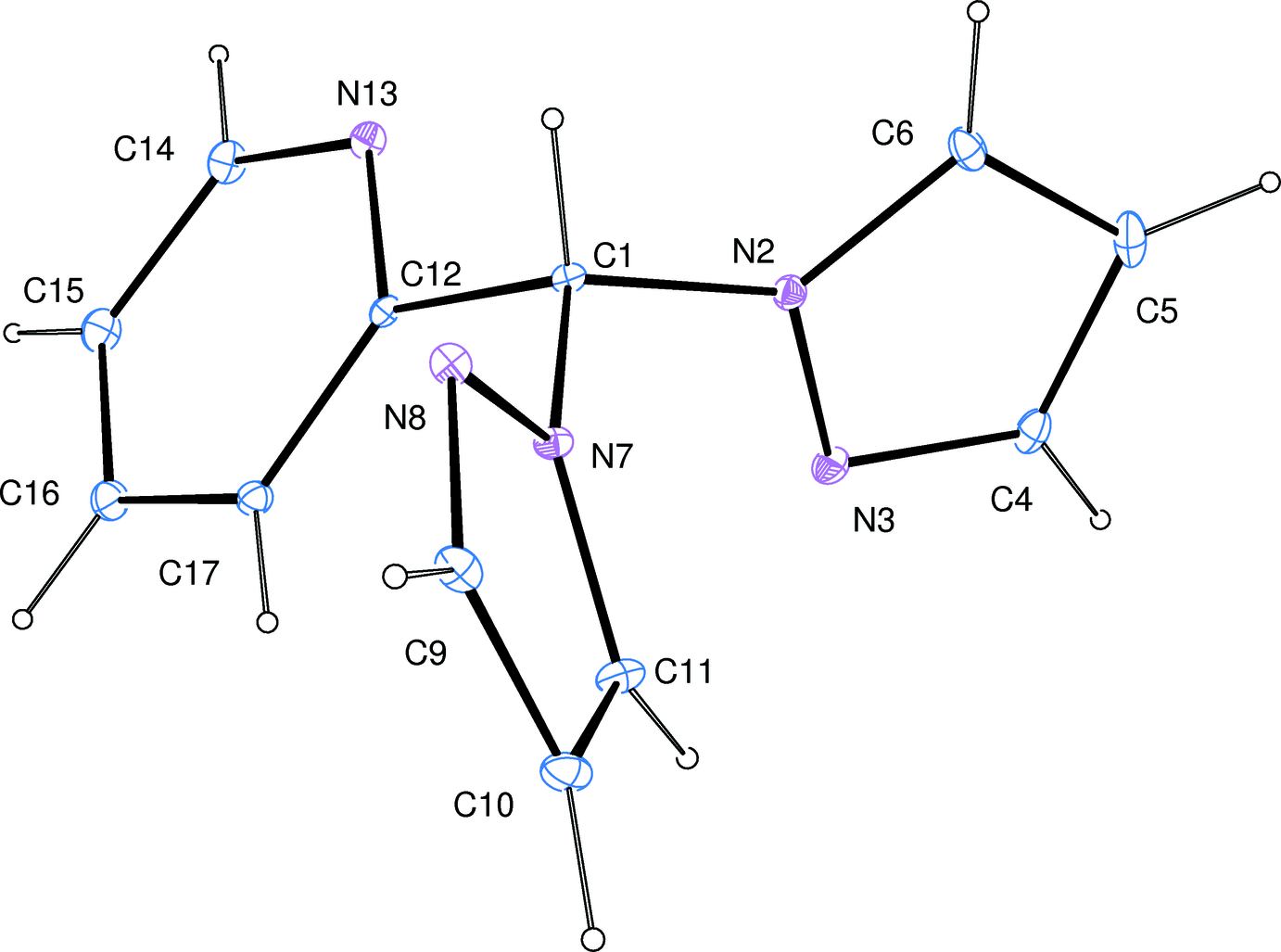

Supplement: Supplementary file 4 [file e-71-0o567-fig1.tif]

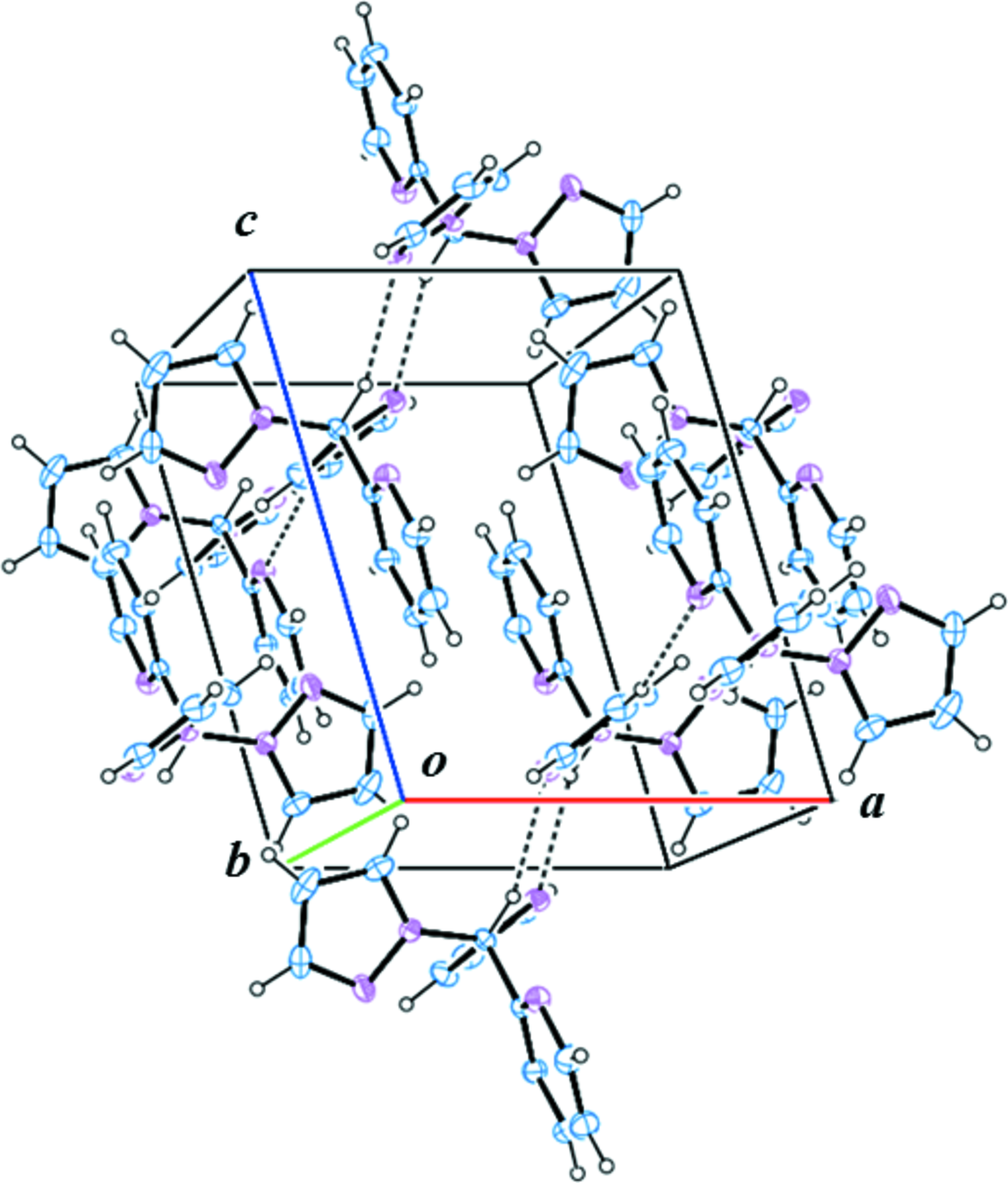

Supplement: Supplementary file 5 [file e-71-0o567-fig2.tif]
